# An evaluation of processing methods for HumanMethylation450 BeadChip data

**DOI:** 10.1186/s12864-016-2819-7

**Published:** 2016-06-22

**Authors:** Jie Liu, Kimberly D. Siegmund

**Affiliations:** Department of Preventive Medicine, USC Keck School of Medicine, University of Southern California, Los Angeles, USA; Department of Preventive Medicine, USC Keck School of Medicine, 2001 N. Soto Street, Suite 202 W, Los Angeles, CA 90089 USA

**Keywords:** HumanMethylation450 BeadChip, Preprocessing, Normalization, Batch correction, Concordance plot

## Abstract

**Background:**

Illumina’s HumanMethylation450 arrays provide the most cost-effective means of high-throughput DNA methylation analysis. As with other types of microarray platforms, technical artifacts are a concern, including background fluorescence, dye-bias from the use of two color channels, bias caused by type I/II probe design, and batch effects. Several approaches and pipelines have been developed, either targeting a single issue or designed to address multiple biases through a combination of methods. We evaluate the effect of combining separate approaches to improve signal processing.

**Results:**

In this study nine processing methods, including both within- and between- array methods, are applied and compared in four datasets. For technical replicates, we found both within- and between-array methods did a comparable job in reducing variance across replicates. For evaluating biological differences, within-array processing always improved differential DNA methylation signal detection over no processing, and always benefitted from performing background correction first. Combinations of within-array procedures were always among the best performing methods, with a slight advantage appearing for the between-array method Funnorm when batch effects explained more variation in the data than the methylation alterations between cases and controls. However, when this occurred, RUVm, a new batch correction method noticeably improved reproducibility of differential methylation results over any of the signal-processing methods alone.

**Conclusions:**

The comparisons in our study provide valuable insights in preprocessing HumanMethylation450 BeadChip data. We found the within-array combination of Noob + BMIQ always improved signal sensitivity, and when combined with the RUVm batch-correction method, outperformed all other approaches in performing differential DNA methylation analysis. The effect of the data processing method, in any given data set, was a function of both the signal and noise.

**Electronic supplementary material:**

The online version of this article (doi:10.1186/s12864-016-2819-7) contains supplementary material, which is available to authorized users.

## Background

DNA methylation, featured by the presence of 5-methylcytosine in the context of CpG dinucleotides, is the most studied form of epigenetic modification. It plays an important role in both physiological processes and disease states. For instance in cancers, alterations in DNA methylation landscapes include a global hypomethylation of the genome accompanied with CpG island hypermethylation [1]. DNA methylation alterations are also described for other types of diseases, such as neurological and autoimmune diseases, and other disorders such as cardiovascular diseases, metabolic diseases and myopathies [2]. Furthermore, epidemiological studies have revealed associations between DNA methylation and exposures such as prenatal maternal smoking [3] and environmental chemicals [4]. The Illumina Infinium HumanMethylation450 (HM450) BeadChip is a popular technology for large-scale DNA methylation profiling due to its advantage in reagent cost and time, comprehensive coverage and high throughput.

Along with the widespread application of the HM450 array, a number of statistical approaches have arisen to address technical noise in the estimate of DNA methylation level. For a targeted cytosine, probes bind to methylated and unmethylated alleles and emit a fluorescence signal. The DNA methylation level, called Beta value, is estimated by the ratio of the methylated to the sum of methylated and unmethylated intensities. Issues encountered during probe design resulted in the implementation of two types of chemical assays, Infinium I and Infinium II, with different technical characteristics. The Infinium type I design utilizes two probes in the same color channel to quantify methylated and unmethylated alleles, the color channel determined by the nucleotide 5’ to the target cytosine (green for G/C and red for A/T); type I probes are more likely to target CpG-dense regions and in the body of the probes all CpGs are assumed to have the same methylation state as the target site. In contrast, the Infinium type II design utilizes one probe but two color channels for a single cytosine target, with green and red channel measuring methylated and unmethylated alleles, respectively; type II probes contain a degenerate base at the cytosine position for CpGs in the probe body. Technical noise is introduced by the variation in background fluorescence signal across arrays and color channel and the different average intensities in the red and green channels. Together, these can introduce additive and multiplicative errors to the signal intensity, reducing the dynamic range of the Beta value and skewing the values of Infinium II Beta values differentially across samples. Other studies showed type II Beta values have lower dynamic range than type I Beta values [5, 6] and that technical variation potentially varies by position on the chip (12 samples/BeadChip), and processing time or place.

Given the increasing popularity of the HM450 array and the biases observed due to platform design, several approaches and pipelines have been developed, either targeting a single issue or designed to address multiple biases within a complete framework. Recently, some systematic comparisons of preprocessing procedures have appeared [7–10], however, evaluations of combination approaches are less common [11, 12]. Pidsley et al. [13] evaluated the combining of methods with different mechanisms together. However, improved approaches have since appeared and are worthy of further consideration. Of interest is (1) the overall best approach for signal processing; (2) the performance of recent between-array methods when analyzing data with substantial biological heterogeneity; and (3) the efficacy of processing approaches when DNA methylation differences are subtler than the stark changes observed in tumorigenesis or aging. In this study nine processing methods are applied and compared in four datasets. The methods include both between-array and within-array normalization methods, as well as combination approaches that sequentially apply procedures addressing different platform biases. The datasets range from cancer data to Alzheimer’s brain data, showing distinctly different variation in DNA methylation. The methods are evaluated based on their ability to reduce technical variance, as well as to identify reproducible differential methylation positions (DMPs) in case-control studies. We show that the performance of processing methods will depend on the nature of the datasets, and, in general, within-array processing for background and probe design bias performs well in all datasets. Furthermore, we reveal that the within-array methods appear robust to obtaining reproducible results across different types of data sets. This should be especially meaningful when dealing with clinical research data where we have only one sample per group. Finally, between-array normalization helps when the variation due to noise is greater than the variation due to biological signal; however, when the methylation alterations are substantial in size and number some between-array methods might remove biological signal.

## Methods

### Preprocessing methods

In total we evaluate and compare nine preprocessing approaches based on the following within-array and between-array methods: (1) background correction and dye-bias equalization (Noob) [14]; (2) beta-mixture quantile normalization (BMIQ) [6]; (3) subset-quantile within-array normalization (SWAN) [15]; (4) background adjustment followed by between-array normalization performed separately by probe design (Dasen) [13]; (5) subset-quantile normalization (SQN) [11, 16]; and (6) functional normalization (Funnorm) [17]. All analyses are implemented in R version 3.0.3, Bioconductor version 2.12 [18] with functions Noob, SWAN, SQN and Funnorm implemented in the minfi package (version 1.6.0) [16], and Dasen and BMIQ in wateRmelon (version 1.0.3) [13]. In addition to these published methods, we also combine methods that correct for different biases: (1) Noob + BMIQ; (2) Noob + SWAN; (3) Funnorm + BMIQ. We note that the function Funnorm already includes Noob as a first step.

Briefly, Noob performs within-array normalization correcting for background fluorescence and dye bias. It fits a normal-exponential convolution model to estimate the true signal conditional on the observed intensities, capitalizing on the unique design of the Infinium I probe pairs to estimate non-specific signal from the ‘out-of-band’ intensities, the wavelength in the opposite color channel to their design (*n* = 92,596 for Cy3 features and *n* = 178,406 for Cy5 features). These background-corrected intensities are then normalized for variation in average intensity in the red and green channel via a multiplicative scale factor computed using the average intensities of the positive control probes.

BMIQ is a mixture-model-based normalization method designed to correct the type II probe bias and make the methylation distribution of type II features comparable to the distribution of type I features. BMIQ fits a three-state (unmethylated, 50 % methylated and fully methylated) beta mixture model for the type I and type II probes separately, with probes assigned to the state with maximum probability. Beta values for the type II features are normalized by state to the distributions of the same estimated in type I features. Like Noob, it is a within-array method.

SWAN is also a within-array normalization method for probe type design, but unlike BMIQ, it starts from methylated and unmethylated intensities. SWAN is based on the assumption that probes with the same number of CpGs in the probe body should have similar intensity distributions (since the regions they interrogate should have similar biological features). For the methylated and unmethylated intensities separately, a random subset of type I and II probes matched on underlying CpG number are selected and quantile normalized. The intensities of the remaining probes are adjusted by linear interpolation. Consequently, the intensity distributions between two probe types are identical in each subset, while the difference in overall distributions between two probe types still remains.

In contrast to the first three methods, the Dasen method is a between-sample normalization method, and like Noob and SWAN, it adjusts the raw intensities instead of Beta values. The background of type I probes is adjusted to match that of the type II and standard quantile normalization applied to methylated and unmethylated intensities separately by probe type (I or II).

SQN is another between-sample normalization method for methylated and unmethylated intensities that also involves within-sample normalization of type I and II probes. Type II intensities are normalized across arrays, and within arrays, type I intensities are normalized to the type II distributions within four feature subsets (CpG island, CpG island shore, CpG island shelf, and Open Sea) ([16]). The stratified within array normalization allows for the biased distribution of type I and II probes by genomic regions, with type I probes appearing more frequently in CpG dense regions that are typically unmethylated.

Funnorm is a between-sample (functional) normalization method that attempts to remove unwanted variation by adjusting for covariates estimated from a control probe matrix. Briefly, 42 summary measures are estimated from the combined 848 control probes and type I ‘out-of-band’ intensities, with the first *m* = 2 principle components of the summarized measures chosen as covariates for intensity adjustment. Adjustment is performed separately in methylated and unmethylated intensities, and in type I and II probes. For probes mapped to X and Y chromosomes, males and females are processed separately, with ordinary quantile normalization used for probes on the Y chromosome because of the small number of probes (*N* = 416). By default the functional normalization is applied after Noob in the current version of minfi package (version 1.6.0).

The summary of data preprocessing methods used in this study is shown in Additional file 1. We note that both SQN and Funnorm normalize features on X and Y chromosomes in males and females separately, while Dasen does not. Also, Funnorm only uses control probes for between-array normalization whereas SQN and Dasen normalize signals using the biological features directly.

### Illumina HumanMethylation450 data sets

Five datasets are used to evaluate the different processing methods. One contains six technical replicates from human peripheral blood lymphocytes (PBLs), and is used to investigate how the processing methods reduce technical variances. These samples were commercially bought, pooled human PBLs, and are the same as used in [14]. The second data set is five lung adenocarcinomas from The Cancer Genome Atlas (TCGA), measured using both HM450 and whole-genome bisulfite sequencing (WGBS) platforms. These data, shared by Titus et al. [19], allow us to benchmark our DNA methylation estimates for HM450 with a whole-genome sequencing-based assay. The remaining three datasets are used to evaluate reproducibility of differential methylation analysis with data processing. The motivation is that better signal processing should lead to better reproducibility in identifying differential methylation. Two of the latter datasets are from TCGA with large-scale methylation differences between cancer and normal tissue, and one is from an unpublished brain data set, showing subtle methylation differences between cases with Alzheimer’s disease and controls.

All samples are analyzed using the Infinium HumanMethylation450 BeadChip, and DNA methylation levels are reported as Beta (β) values, the proportion of methylation intensity to the total intensity, ranging from 0 to 1. The samples from the last three studies are divided into discovery and validation datasets for evaluating reproducibility of testing differential DNA methylation. Each processing method is performed in the discovery and validation set separately. The separate processing will not affect the Beta values for individual samples processed using within-array methods (Noob, SWAN, BMIQ), but can affect the Beta values when using between-array processing methods (Dasen, SQN, Funnorm). The details for defining discovery and validation data sets are described below, and summarized in Additional file 2. All samples are anonymized, and this study did not require institutional review board approval.

#### The TCGA-KIRC dataset

We use the identical samples and study design implemented by Fortin et al. [17]. In total, 222 kidney clear cell tumor samples and 160 non-tumor kidney samples are assayed and included in the study. These samples are split into discovery (training) and validation (testing) datasets. The discovery set contains 65 tumor samples and 65 non-tumor kidney samples, analyzed in two plates with little variation between plates. The validation set contains 157 tumor samples and 95 non-tumor kidney samples, spread over 4 plates and designed to have larger variations among samples.

#### The TCGA-COAD dataset

A total of 321 COAD (colon adenocarcinoma) samples (284 tumor/37 non-tumor colon) are included in this study. These samples are selected by plate to create discovery and validation datasets. No substantial plate-to-plate variation was observed in COAD dataset (Additional file 3). The discovery set was assigned 143 tumor samples and 17 controls spread across 7 plates, and the validation set 141 tumors and 20 controls run over 4 plates. All samples had fewer than 5 % missing Beta values in each color channel and were analyzed on plates with more than 2 COAD samples.

#### The Brain data

A total of 376 bulk brain samples obtained from the middle temporal gyrus were analyzed over 8 plates. Of these, 215 are from patients diagnosed with Alzheimer’s disease (AD) and 161 are from controls, frequency matched by age and sex. Once more, discovery and validation data sets are created selecting samples by plate. A total of 180 samples across the first four plates are assigned to the discovery set (102 diseased and 78 controls) and 196 samples from the latter four plates to the validation set (113 diseased and 83 controls). These data were generated at the University of Southern California.

### Evaluation metrics

Improved measures of DNA methylation should increase our power to detect biological associations. However, evaluating our ability to detect true signals in real data is complicated by the fact that we do not know which signals are genuine. However, we can study the reproducibility of differential methylation results between different data sets. Higher reproducibility would reflect more potential for a method in revealing true signals, while poorer agreement indicates the results are more likely due to chance. We assess differential methylation by disease status for the case-control data by applying two-sample t-tests separately in discovery and validation sets. The data are analyzed on the Beta-value scale and tests are two-sided. Several tests of reproducibility are performed. First, result reproducibility is evaluated from the lists of (ranked) *p*-values using concordance plots and ROC curves. Second, we assess overlap of differentially methylated positions (DMPs) applying the Benjamini-Yekutieli method [20] to control the false-discovery rate (FDR-adjusted *p* <0.05). Benjamini-Yekutieli allows for correlation in test results and results in many fewer significant test results than methods that ignore correlation between test statistics.

#### Concordance plot

In a concordance plot, the overlap percentage is plotted against feature list size for lists of size one to k, where the overlap percentage is defined by the proportion of features ranked in the top k in both the discovery and validation set. The processing approach with higher overlap percentages across different list sizes indicates higher reproducibility. If the data set is underpowered and the results are not reproducible, the overlap percentage follows a diagonal line through the origin, with slope equal to the inverse of the number of features. This approach has connections with the change of correspondence plot proposed for the irreproducibility rate framework [21].

#### ROC curves

ROC curves are used to quantify, for a known gold standard, the true positive and false-positive results using all possible threshold values of a quantitative marker. Although the genuine gold standard is unknown, we use the results from the discovery subset, samples selected because they reflected lower technical variation between plates, to define the ‘gold standard’, and pick the number of true signals from a list size with high overlap percentage from the concordance plots. Specifically, for the TCGA KIRC and COAD data sets, the features from the top 100,000 ranked *p* values in the discovery subset are defined as “true signal”. For the brain data, we anticipate small differences and only select the top 100 in the discovery set as “true signals”. These same list sizes are used in [17] for evaluation of strong and weak signals. The area under the curve (AUC) is computed to assess the performance of a method. A method with good reproducibility will have an ROC curve above the diagonal line, with high AUC. The ROC analysis is performed using ROCR package in R (version 1.0-7).

### Feature filtering

A total of 485,512 cytosines are queried by the HM450 BeadChip array. For method evaluation, we filter probes with missing Beta values across samples. For the TCGA and brain data sets we also exclude probes mapped to the X or Y chromosomes, containing a common SNP (minor allele frequency > 0.1) within 10 bp of the target cytosine, or map to multiple regions of the genome. This filtering results in 485,110 features in the PBL dataset, 371370 in KIRC, 384470 in COAD, and 360894 in the brain data set.

It is worth noting that the minfi and methylumi packages in R have different criteria in assigning missing to Beta values. In minfi, missing values (“NAs”) are assigned when both the raw methylated and unmethylated intensities are zero. Although this catches all features with failed probes that do not fluoresce, it allows features fluorescing at background levels for both M and U probes to have Beta values near 0.5. We favor the additional assignment of ‘NA’ to features that do not have at least one of the M and U intensities fluoresce above background. Such masking is applied in the methylumi package, where the detection *p* values are used for determining missing Beta values; the detection *p*-value reports the quantile from the distribution of 600 negative control probes (from the same color channel) for the larger of the methylated or unmethylated intensity. Then, the Beta value will be assigned missing if the corresponding detection *p*-value is more than 0.05 (not above background). This can result in a much larger number of missing Beta values than assigned in the minfi package. We use missing values assigned by the methylumi package.

## Results

### Reduction in technical variance

**Fig. 1:**
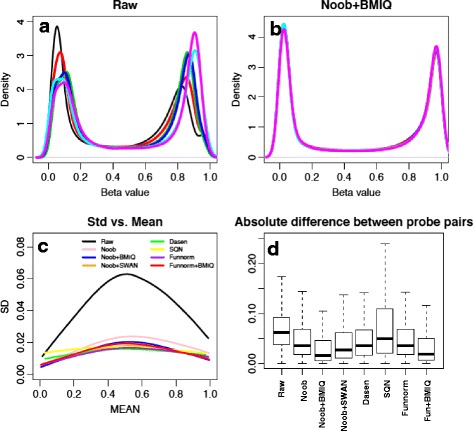
Evaluation of preprocessing methods in reducing technical variance using PBLs dataset. **a** Density distribution of Beta values in six replicates in the raw data. **b** Density distribution of Beta values in six replicates in the data processed by Noob + BMIQ. **c** Loess curve fitted to the scatterplot of standard deviation versus average of methylation Beta values for different processing methods. **d** Boxplot of absolute difference in methylation Beta values between adjacent type1-type2 probe pairs in PBLs dataset, averaged across six replicates

We assess the ability of preprocessing methods to reduce technical variance and adjust type I/II bias using six technical replicates from commercially available pooled human male peripheral blood lymphoctytes (PBLs) assayed as part of a larger experiment [22] and previously evaluated in [14]. Figure 1a shows variability in the density distributions of the Beta values for the six replicates that is no longer evident after processing the data using Noob + BMIQ (Fig. 1b). The density distributions, stratified by probe type, appear in Additional file 4. For the raw data the density distributions vary considerably across the six replicates, especially among type II probes; the reduced dynamic range of raw Beta values for type II probes relative to type I probes was also apparent [5]. All of the normalization methods increase the dynamic range of the type II probes with perhaps the greatest reduction in type I/type II bias seen when combining Noob with BMIQ. It is worth of noting that SQN changes the distribution of type I probes most noticeably, presumably because it uses type II probes as the anchors when normalizing between two probe designs in each sub-category (defined by genomic context relative to CpG islands).

Figure 1c shows a smoothed curve summarizing a scatterplot of standard deviation versus mean Beta values across six replicates for all probes. All processing methods reduce the average standard deviation compared to the raw data. The correlation between standard deviations and means is least obvious after application of Dasen and SQN, possibly due to the fact that both Dasen and SQN are between-array methods that normalize methylated and unmethylated intensities separately. Interestingly, the within-array methods are competitive with the between-array methods despite the application to technical replicates for which all between-array assumptions are met. Also, Funnorm shows good stability despite the use of principal components adjustment estimated from only six arrays.

Figure 1d shows a boxplot of the average of the absolute difference in Beta value for the 32,713 pairs of adjacent type I/II probes that lie within 200 bp of each other. Based on the well identified spatial correlation of DNA methylation at scales < 500 bp [23], we expect that the ideal normalization algorithms should minimize the averaged absolute difference, as previously observed by Teschendorff et al [6]. Noob + BMIQ seems to show the greatest reduction in deviation between measures from adjacent type I/type II probes, while SQN does not perform well with respect to reducing type I/type II bias.

### Cross-platform comparison with whole-genome bisulfite sequencing

**Fig. 2:**
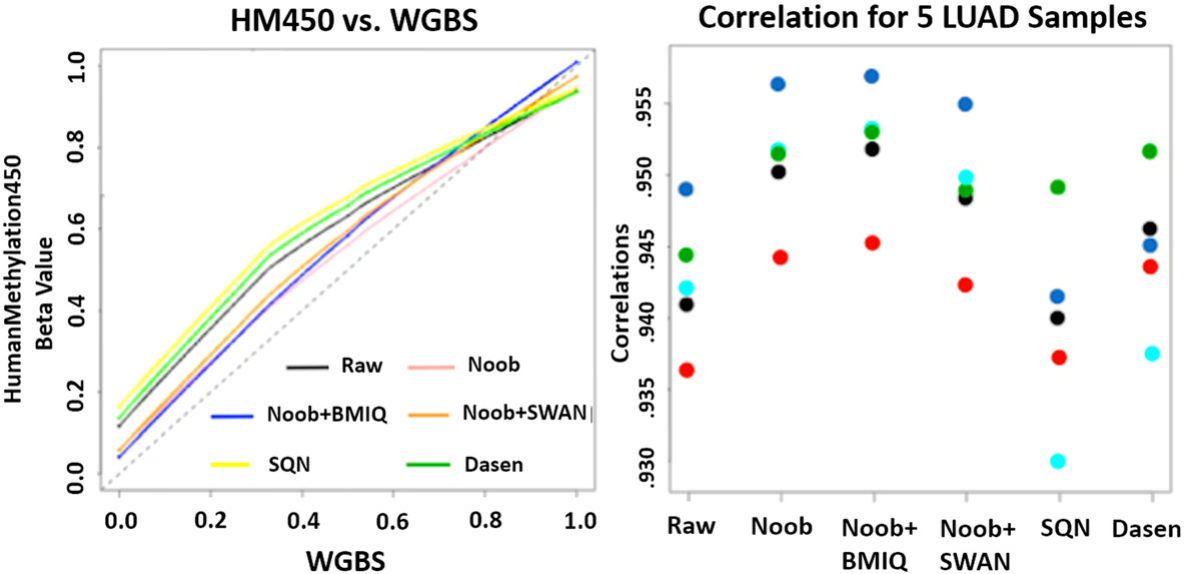
Cross-platform comparison of Beta values from HumanMethylation450 vs whole-genome bisulfite sequencing. **a** For 1 LUAD sample, loess curve fitted to 199441 paired Beta values for WGBS and HM450 data after different processing methods. The dashed line indicates equal values, **b** Scatter plot of correlations between WGBS Beta values and HM450 Beta values after different processing methods in 5 LUAD samples. Different colors represent different samples. Aqua shows the results from the sample in **a**)

We used five lung adenocarcinoma (LUAD) samples from TCGA to benchmark the post-processing HM450 Beta value estimates to estimates from WGBS. The number of features available for cross-platform comparison ranged from 110,962 to 199,441 after restricting to cytosines with a minimum sequencing depth of 10. Loess curves summarizing the relationship between the Beta values from the two platforms showed Noob + BMIQ estimates were most similar to WGBS (Fig. 2a). The Pearson correlation was highest for Noob + BMIQ (0.953), lowest for SQN (0.930) and Dasen (0.937), and intermediate for the raw data (0.942). The other four LUAD samples also showed the highest Pearson correlations for Noob + BMIQ processed data (Fig. 2b).

### Reproducibility of differential DNA methylation analysis

**Fig. 3:**
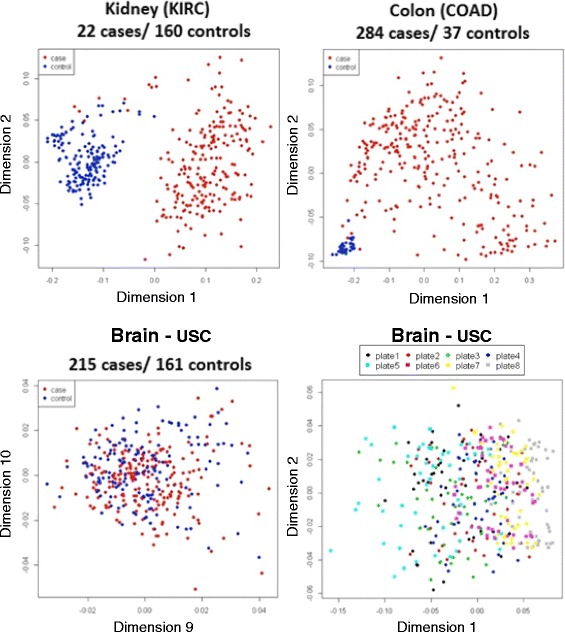
Multidimensional scaling plot of distances between samples in three datasets. **a** TCGA-KIRC, **b** TCGA-COAD, **c** Alzheimer’s brain data, scaling dimensions 9 vs 10, **d** Alzheimer’s brain data, scaling dimensions 1 vs 2, samples coloured by plates. Euclidean distances between sample pairs are computed for a common set of 5000 features having the largest standard deviations across all samples. Features containing SNPs or mapping to the sex chromosomes were excluded. Samples are colored by disease status (**a**,**b**,**c**) or plates (**d**)

The ability to reliably identify features differentially methylated by disease subtype will depend on the strength of the biological signal and noise due to technical issues. A multidimensional scaling plot of the top 5000 variable features allows us to visualize sample distances by disease state for our three studies (Fig. 3). For the KIRC and COAD data sets, the first dimension explains 49.8 and 43.7 % respectively, of the total variance in Beta values from the raw signals, with the second dimension explaining only 5.9 and 6.0 % (Fig. 3a, b). These two scaling dimensions allow us to visualize a clear separation between cancer and control samples for both the discovery and validation data sets indicating a large biological signal even in the raw data. On the other hand, the first and second scaling dimensions for the brain data only explain 29.9 and 3.8 % of the total variance, and it is not until the 18^th^ dimension that the cumulative proportion of variance exceeds 50 %. Case-control differences are not apparent until the 10^th^ dimension (t-test *p* = 0.002) (Fig. 3c). Figure 3d shows scaling dimensions 1 and 2 for the brain data with samples colored by processing plate. We see that the 1^st^ scaling dimension is associated with sample plate, with plate 5 and plate 8 samples appearing more towards the right side of the figure.

**Fig. 4:**
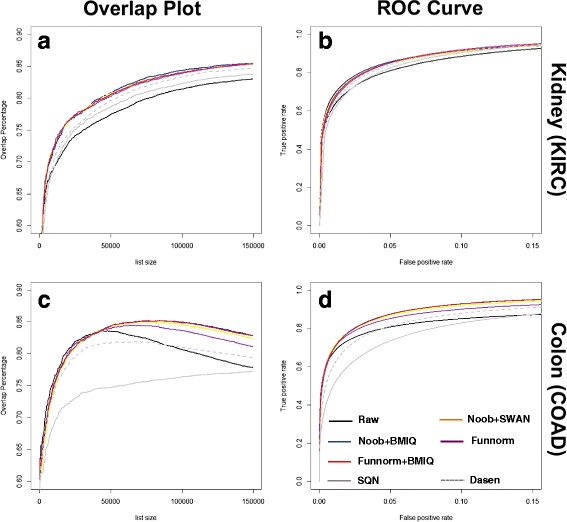
Overlap plot and ROC curves for KIRC (**a**,**b**) and COAD (**c**,**d**) datasets. The top ranked 100,000 features in the discovery set were defined as genuine signals for the ROC curve analysis (**b**, **d**)

We use concordance plots to present the overlap in the top ranking DMPs as a function of list size when applying the different processing methods. Combining the methods Noob with BMIQ or SWAN always showed higher reproducibility than any of the three methods alone so the results for the individual methods are not shown. For the two cancer data sets, all within-array combination methods show similar performance to one another and all show higher concordance compared to the raw data (Fig. 4a and c). The performances of between-array methods show more variability. Funnorm, a method that utilizes control probes for normalization, always performs better than the raw data, but not better than the within-array methods. SQN and Dasen, two between-array methods that only use signal from the biological features for normalization, only perform better than the raw data for the KIRC study; for COAD, they are worse (*Grey lines* in Fig. 4a, c). The poor performance might be due to the substantial heterogeneity among the COAD tumor samples relative to control samples (as is shown in Fig. 3b), and normalizing intensities across samples may hide true biological signals. In contrast to the concordance plot which reflects sensitivity across different list sizes, the ROC curve shows sensitivity across different false positive rates for a specific list size. In our study the results of ROC curve are consistent with the overlap plot analysis, showing high sensitivity for Noob + BMIQ, Noob + SWAN, and Funnorm + BMIQ (Fig. 4b, d with a, c, respectively). And whereas there is little variation in sensitivity among the evaluated methods for the KIRC study, the COAD study shows a clear loss in sensitivity when performing between-sample normalization using SQN (Fig. 4d).

We note that for each of these two cancer datasets, the concordance after processing nears 85 %. However, the concordance of discoveries is above 70 % even for the raw data at a list size of 15,000 features. The overlap percentage for the raw COAD data is over 80 % for the top 50,000 features. These results are not surprising given that the separate clustering of cancer and non-cancer samples in the MDS plots reflects substantial variation in DNA methylation between groups with genuine signals very likely to be detected and reproduced. The higher homogeneity of colon control samples and their homogeneity relative to cancer samples might explain the higher reproducibility of DMPs in the COAD study (see Fig. 3b).

**Fig. 5:**
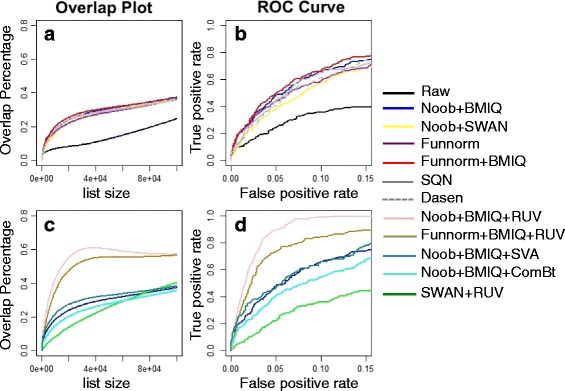
Overlap plot and ROC curves for brain dataset without batch correction (**a**,**b**) and with batch correction (**c**,**d**). The top ranked 100 features in the discovery set were defined as genuine signals for the ROC curve analysis (**b**, **d**)

Reproducibility for the Brain data is much lower than for the cancer data, which was not surprising either based on the earlier MDS figures. This time, the fraction of overlapping discoveries from the analysis of the raw data appears essentially random (Fig. 5a); however, the processed data sets are able to show a small enrichment in the overlap of top hits. This time the greatest overlap occured for the between-array method Funnorm combined with (within-array) BMIQ. The advantage over Noob + BMIQ, the second best method, was modest. We note that the strictly within-array methods Noob + BMIQ or Noob + SWAN appeared as good as or better than the between-array (Noob+)Funnorm alone, showing the importance of correcting for probe design bias. This time the between-array methods SQN and Dasen were always much better than the raw data, but never achieved the reproducibility of the top within-array method, Noob + BMIQ. The same results are seen from an ROC curve analysis using the top 100 features as the gold-standard (Fig. 5b). All together, these results suggest that correction for technical effects in these data is needed to find the more subtle biological signal. Furthermore, the modest overlap percentage after normalization led us to investigate RUVm, the new variant of remove unwanted variation designed for removing batch effects in DNA methylation data [24]. We evaluated RUVm for batch effect correction, comparing it to competing methods surrogate variable analysis (SVA) [25] and ComBat [26].

RUVm improved result reproducibility, outperforming both SVA and ComBat. In fact ComBat showed a minor loss in reproducibility over no batch correction, suggesting there was confounding between samples and BeadChips. Applying RUVm to Noob + BMIQ processed brain data resulted in a nearly 30 % improvement in overlap percentage, achieving a max 63 % overlap in the brain data set, up from 36 % observed using normalization methods alone (Fig. 5c). The results using RUVm depended heavily on the original data processing, with Noob + BMIQ + RUVm showing a 65 % increase in sensitivity compared to SWAN + RUVm at a 5 % false-positive rate (Fig. 5d).

Lastly, we investigated whether our results are sensitive to the single split of the data into discovery and validation sets. We resampled the brain data ten times into new discovery and validation sets, each time computing the AUC for the raw data, the data processed with Noob + BMIQ, and the data processed with Noob + BMIQ + RUVm. There was very little overlap in distributions, with AUC interquartile ranges (IQR) 0.65-0.68 for the raw data, 0.7-0.82 for Noob + BMIQ and 0.95-0.97 for Noob + BMIQ + RUVm. This order of results held their rank, and suggested that one data split was representative, and the performance does not change with repeated sampling.

## Discussion

In this study nine preprocessing methods for Illumina HM450 array data are applied and compared, using combinations of three within-array and three between-array methods. Since different normalization methods address different technical effects in the data, we take advantage of this diversity of approaches and combine methods addressing different mechanisms.

Analysis of technical replicates showed that different methods optimized different assessment criterion. The within-array methods Noob + BMIQ and Noob + SWAN were favored at removing type I/type II bias, while the between-array methods Funnorm, SQN and Dasen, reduced between-sample variability the best. However, a recent paper by Lemire *et al.* found Noob + BMIQ performed best in reducing differences between Beta values in intra-plate duplicates [27]. SQN, a between-array method that normalizes intensities between typeI/II probes, did the least to remove probe type bias; this might be due to the fact that SQN utilizes type II probes, those known to show greater bias, as “anchors” when normalizing type I intensities. The results consistently showed that *a priori* background correction and dye-bias normalization using Noob improved both bias and variance over type I/type II correction methods alone (BMIQ and SWAN). Analysis of paired HM450 and WGBS data showed the highest Pearson correlations after using Noob + BMIQ to process the HM450 data.

When evaluation focused on detecting reproducible DMPs across different disease data sets, within-array normalization and between-array methods that relied only on control features consistently displayed the highest reproducibility. The performance of between-array methods that utilized biological features for normalization depended on the characteristics of the data set. When disease state was not associated with the principal scaling dimension, between-array methods tended to improve sensitivity of reproduced signals. Still, they never outperformed the best within-array methods. Furthermore, when the biological signal was strong, they had the potential to behave worse than no processing at all. This was the case for the COAD data set for which the first two principal scaling dimensions were both associated with disease status. It also agreed with the separate finding of a lower validation rate after SQN by Wu *et al.* [9]. Overall, we found the within-array method Noob + BMIQ to consistently provide the most reproducible signal detection across the three data sets.

We evaluated three batch-correction methods capable of removing additional variation not accounted for by data processing (RUVm, SVA, and ComBat), to see whether they could improve the low reproducibility of DMPs in the brain data. Both RUVm and SVA improved reproducibility, however using ComBat to adjust for BeadChip effects reduced it. This suggested that there was confounding of case-control comparisons by BeadChip. Application of RUVm increased DMP reproducibility in the brain data the most, and interestingly, a large variation in performance appeared depending on the preprocessing method applied; Noob + BMIQ turned in substantially superior reproducibility compared to competing approaches.

Finally, Beta regression has been proposed as a more powerful test of differential DNA methylation than t-tests [28]. However, since current software for Beta regression is slow, and our focus was on finding the most reproducible processing method instead of the most sensitive test, we used t-tests on the Beta values. Data that are subjected to variance-stabilizing transformations prior to t-tests might show higher sensitivity for differential testing [28], with the difference (in sensitivity) a function of both the effect size and sample size. The already high overlap percentages observed in our cancer studies suggest that data transformation is unlikely to change the results. For the brain data, it is possible that data transformation could improve the overlap percentages for the different normalization methods. The analyses with batch correction were performed on the logit scale, so these potentially represent the most sensitive t-test result.

## Conclusions

This study provides a comprehensive comparison of the currently popular normalization methods in processing HM450 array data. Combinations of methods are applied and compared in five data sets, ranging from cancer data to Alzheimer’s brain data, and showing distinctly different variation in DNA methylation. We find that the combination of Noob + BMIQ, a within-array method, performs well in reducing technical variance, adjusting typeI/II bias, and gaining reproducibility in differential methylation analysis. At the same time, the between-array normalization methods might hurt the data when there are global methylation alterations. For differential DNA methylation analysis RUVm was the most powerful batch correction method, and it performed best on data first processed with Noob + BMIQ. The combination of methods and comprehensive comparisons in our study provide valuable insights in processing HM450 BeadChip data.

## Additional files

Additional file 1: Summary of preprocessing methods used in this study. (XLSX 44 kb) Additional file 2: Number of samples in TCGA-KIRC, TCGA-COAD, and Alzheimer’s disease brain data assigned to Discovery and Validation data sets by disease status and plate. (XLSX 45 kb) Additional file 3: Multidimensional scaling plot of distances between samples in COAD, scaling dimensions 1 vs 2, samples colored by plates. Euclidean distances between sample pairs are computed for a common set of 5000 features having the largest standard deviations across all samples. Features containing SNPs or mapping to the sex chromosomes were excluded. (PDF 7 kb) Additional file 4: Density distributions of Beta values for the Type I (solid lines) and Type II (dashed lines) probes for six PBLs replicates under different processing methods. (PDF 50 kb)
