# Supplementary figures and images for: An evaluation of processing methods for HumanMethylation450 BeadChip data
Source: BMC Genomics. 2016 Jun 22;17:469. doi: 10.1186/s12864-016-2819-7 (PMC4918139; doi:10.1186/s12864-016-2819-7)

MDS plot for COAD

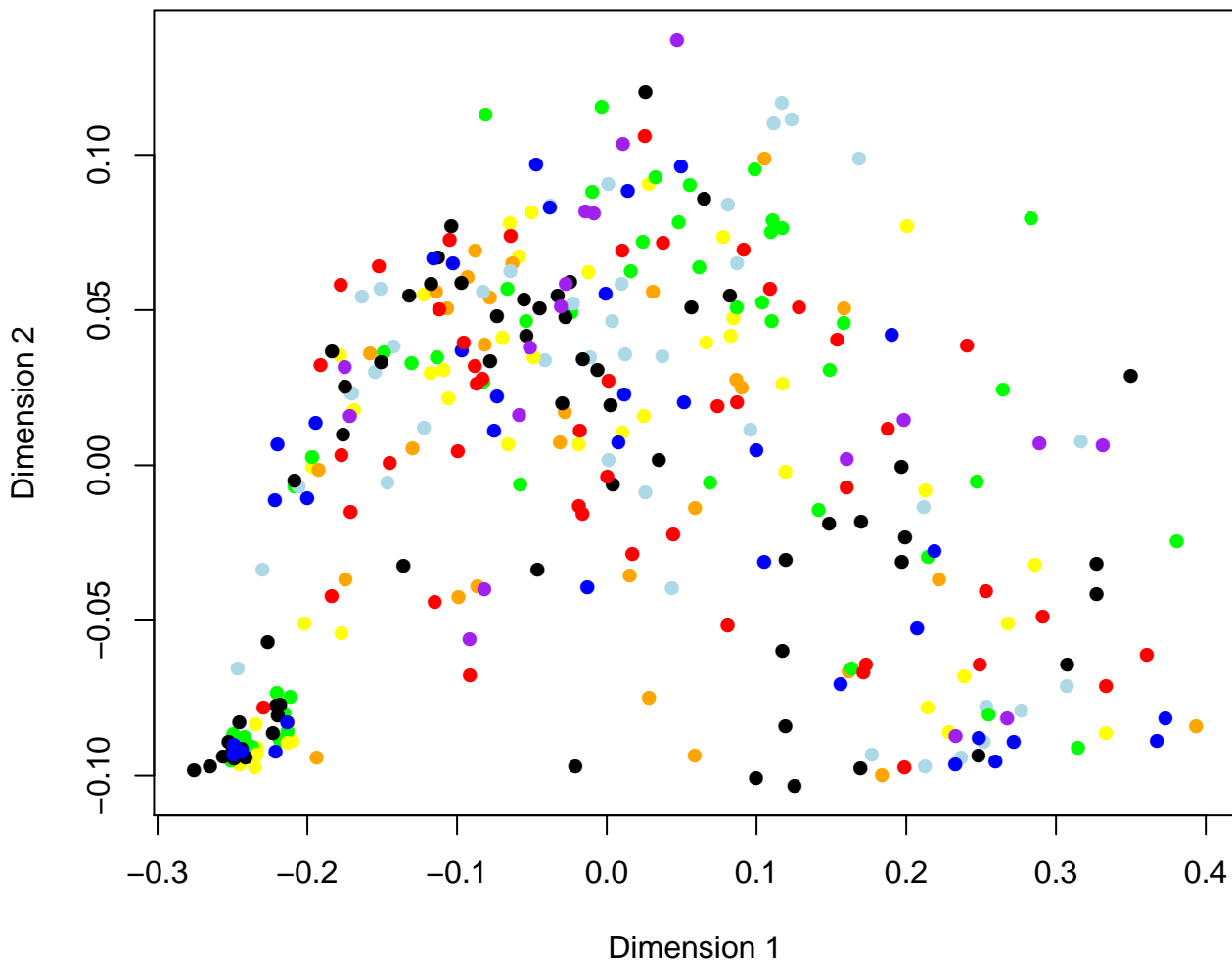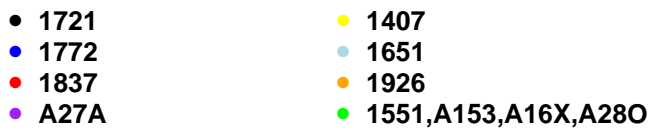

Supplement: Additional file 3: — Multidimensional scaling plot of distances between samples in COAD, scaling dimensions 1 vs 2, samples colored by plates. Euclidean distances between sample pairs are computed for a common set of 5000 features having the largest standard deviations across all samples. Features containing SNPs or mapping to the sex chromosomes were excluded. (PDF 7 kb) [file 12864_2016_2819_MOESM3_ESM.pdf]

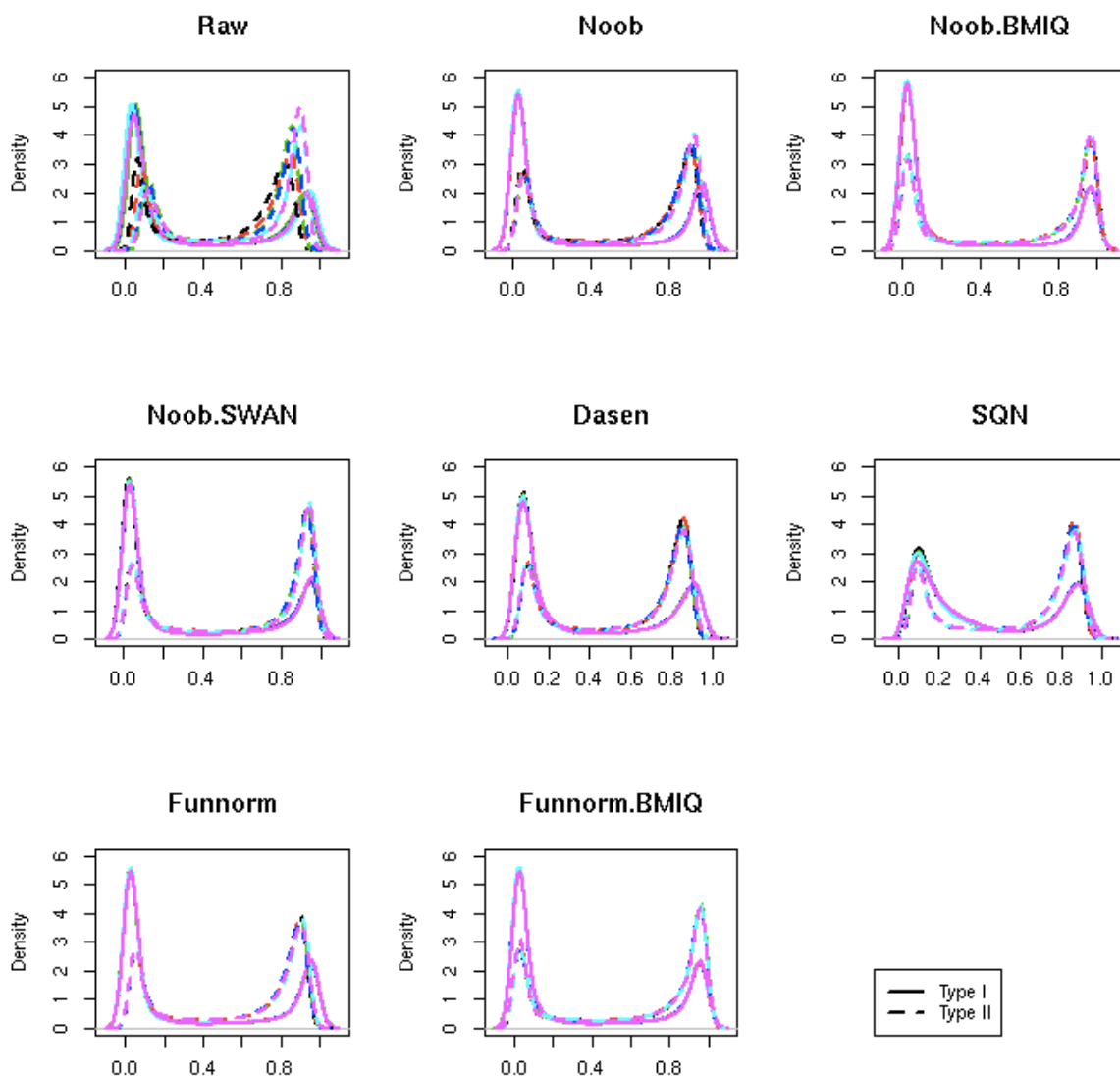

Supplement: Additional file 4: — Density distributions of Beta values for the Type I (solid lines) and Type II (dashed lines) probes for six PBLs replicates under different processing methods. (PDF 50 kb) [file 12864_2016_2819_MOESM4_ESM.pdf]
